# Reporting of determinants of health inequities and participant characteristics in randomized controlled trials of systemic lupus erythematosus in Canada: A scoping review

**DOI:** 10.1177/09612033241233032

**Published:** 2024-02-09

**Authors:** Megan Thomas, Vanay Verma, Niloofar Gheshlaghi, John Esdaile, Antonio Avina-Zubieta, Cheryl Barnabe, Mark Harrison, Mary A De Vera

**Affiliations:** 1 Faculty of Pharmaceutical Sciences, 8166 University of British Columbia, Vancouver, BC, Canada; 2 Collaboration for Outcomes Research and Evaluation, Vancouver, BC, Canada; 3 258853 Arthritis Research Canada, Vancouver, BC, Canada; 4 Department of Medicine, 70401 University of Calgary, Calgary, AB, Canada; 5 Centre for Advancing Health Outcomes, Vancouver, BC, Canada

**Keywords:** Equity, diversity, and inclusion, systemic lupus erythematosus, scoping review

## Abstract

**Objective:**

To report participant characteristics relevant to identifying health inequities in systemic lupus erythematosus (SLE) randomized controlled trials conducted in Canada.

**Methods:**

We conducted a scoping review by searching MEDLINE (Ovid) and Embase (1990 to June 2023), and CENTRAL (inception to June 2023). Eligible studies: used an RCT design; evaluated interventions (pharmacologic and non-pharmacologic) among SLE patients aged ≥18 years; and were conducted in Canada. Data extraction was guided by the Campbell and Cochrane Equity Methods Group’s PROGRESS-Plus framework on 11 factors leading to health inequities (Place of residence; Race, culture, ethnicity, and language; Occupation; Gender and sex; Religion; Education; Socioeconomic status; Social capital; Plus: Personal characteristics associated with discrimination; Features of relationships; and Time-dependent relationships).

**Results:**

Of 1901 unique records, 6 met the inclusion criteria. Sex and age were the only PROGRESS factors that were reported in all studies. The majority of participants were female (84.4% to 100%), and mean ages of participants ranged from 42 to 52.3 years. Place of residence, race, education, and social capital were reported in three studies. Socioeconomic status was reported in two studies, and occupation was reported in one study. Religion, features of relationships, and time-dependent relationships were not reported in any included studies.

**Conclusion:**

Limited reporting of determinants of health inequities in RCTs for SLE in Canada suggests the need for reporting standards to support equity, diversity, and inclusion practices in research.

## Introduction

There is an urgent need to address inequities in health created by structural and social disparities. Race, sex and/or gender identity, sexual orientation, socioeconomic status (SES), and religion among others have been documented to result in unequal allocations of power and resources.^1,2^ This unequal allocation can impact health in various ways, such as through one’s ability to access health services. This is particularly important for complex chronic conditions such as systemic lupus erythematosus (SLE), an autoimmune condition with multiple organ targets, as patients require longitudinal access to care to manage disease activity and symptoms and preserve their health, and disease burden is not equally distributed.

The Campbell and Cochrane Equity Methods Group’s PROGRESS-Plus^3^ framework outlines (risk) factors (‘PROGRESS factors’) that lead to inequities in health (i.e. **P** lace of residence; **R** ace, culture, ethnicity, and language; **O** ccupation; **G** ender/sex; **R** eligion; **E** ducation; **S** ocioeconomic status; and **S** ocial capital) Plus additional factors (i.e. Personal characteristics associated with discrimination; Features of relationships; and Time-dependent relationships). The PROGRESS-Plus framework allows for consideration of health inequities in chronic conditions such as SLE. Although SLE can present in any individual throughout their life span, it presents most commonly among females (Sex),^4,5^ and in populations that are not White (Race, culture, ethnicity, and language),^6^ and possibly preferentially occurs across those living in urban locations (Place of residence).^7^ Those who are members of African, Asian, Indigenous, and Hispanic population groups not only tend to develop SLE earlier but also have a more acute disease onset and more severe clinical manifestations.^2,6^ Low socioeconomic status (SES) has been associated with increased SLE disease activity, organ damage, and mortality.^8^ Further, research shows that social networks (Social capital) play an important role in SLE management, with poor social support being linked to higher disease activity and worse mental function.^9^ Thus, understanding the contributors to health inequities in SLE as outlined in the PROGRESS-Plus framework is an initial step to improving access to care and patient outcomes.

Efforts to address health inequities for SLE care and research in Canada have identified that access to care is negatively affected by racism and geography.^10^ To add to these efforts, it is also important to examine how determinants of health inequities have been considered in SLE clinical research in Canada, particularly because research is how effective therapies for SLE are identified. Missing these determinants of health inequities may limit opportunities to intervene or design interventions, understand treatment, or could inadvertently exacerbate disparities by not including all population groups. Though specific determinants of health inequities such as race and ethnicity are often reported in certain countries (e.g. the US^11,12^), to our knowledge, there has been no similar investigation of the reporting of a broad definition of determinants of health inequities in SLE trials conducted in Canada. As such, our objective was to conduct a scoping review of Canadian randomized controlled trials (RCTs) of interventions among people with SLE to characterize participants and identify which, if any, of a broad range of determinants of health inequities are reported, and how, guided by the PROGRESS-Plus framework.

## Methods

### Inclusion criteria

We followed the Arksey and O’Malley framework for conducting scoping reviews. We included studies that: 1) used an RCT design; 2) evaluated interventions, defined as either pharmacological or non-pharmacological treatments or services; 3) included participants with SLE; 4) conducted in Canada; 5) published in English; and 6) published between 1990 and July 2022. These criteria were selected based on our prior scoping reviews of other rheumatic diseases.^13,14^

### Information sources and search

We conducted a search in MEDLINE (Ovid 1990 to June 2023), Embase (Ovid 1990 to June 2023), and CENTRAL (inception–June 2023) (Supplemental Materials). The search strategy was adapted from the sensitivity-maximizing Cochrane Highly Sensitive Search Strategy for identifying randomized trials in MEDLINE (2008 revision). Following best practices, we consulted with a librarian to develop and refine our search.

### Study selection

All retrieved publications were independently screened for eligibility, first by title and abstract (VV) and then by full text (MT and VV). Any records judged eligible for inclusion proceeded to full text review. Discussion between all authors occurred to resolve uncertainty and to achieve consensus about inclusions.

### Data extraction and synthesis of results

We extracted the following data from included studies: authors, title, journal, year, purpose of the study, and study intervention. Of particular interest was the reporting of eleven PROGRESS-Plus factors: 1) Place of residence; 2) Race, culture, ethnicity, and language; 3) Occupation; 4) Gender and sex; 5) Religion; 6) Education; 7) Socioeconomic status; 8) Social capital; 9) Personal characteristics associated with discrimination; 10) Features of relationships; and 11) Time-dependent relationships. For studies that reported PROGRESS-Plus factors, we extracted further details such as how these factors were defined and operationalized, and the distribution of study participants. Since this was a review, we did not recruit participants, and ethics approval was not required.

## Results

### Included studies

**Figure 1.**
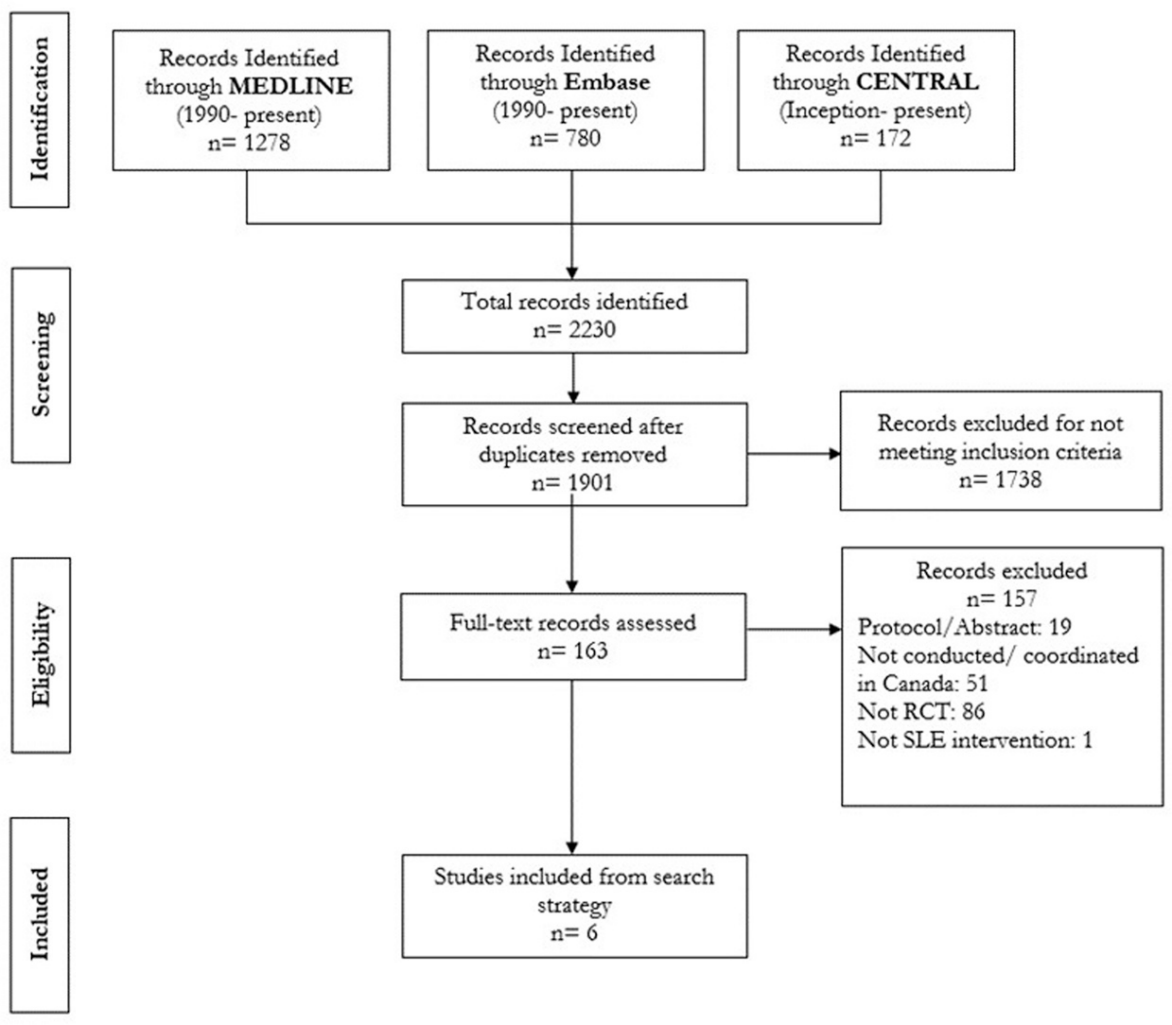
PRISMA.

After screening 1901 unique records, 6 studies were included (Figure 1), with the earliest study being published in 1991, and the most recent in 2023. Of the included studies, 2 evaluated pharmacological interventions^15,16^ and 4 evaluated non-pharmacological interventions^17–20^ (Table 1).

**Table 1.** Characteristics of included studies and reporting of PROGRESS-Plus factors.

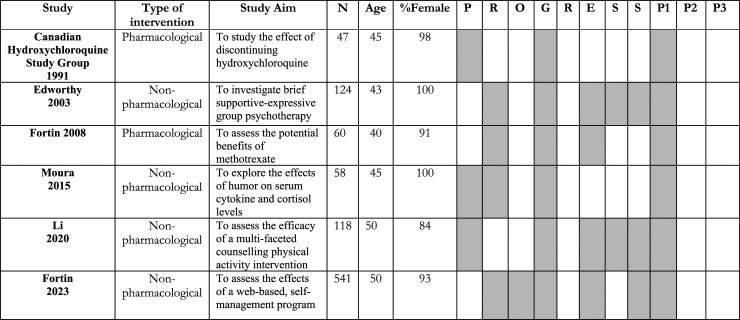

Shaded cells denote factor reported in the study and blank cells denote factor not reported; PROGRESS-Plus Factors: **P** (Place of residence), **R** (Race/ethnicity/culture/language), **O** (Occupation), **G** (Gender/sex), **R** (Religion), **E** (Education), **S** (Socioeconomic status), **S** (Social Capital), **P1** (Personal characteristics associated with discrimination), **P2** (Features of relationships), and **P3** (Time-dependent relationships).

### Characteristics of participants

All included studies reported sex and age (i.e. personal characteristics associated with discrimination). Participants in included RCTs were predominantly female (84% to 100% of participants in included studies). Reported mean ages of participants ranged from 40.2 (IQR 34.0–48.2) years to 52.3 (SD 14.1) years, which suggests individuals were largely middle-aged. Participants were predominantly White (72.4% to 84% of participants when reported).

### Reporting of progress-plus factors

**Figure 2.**
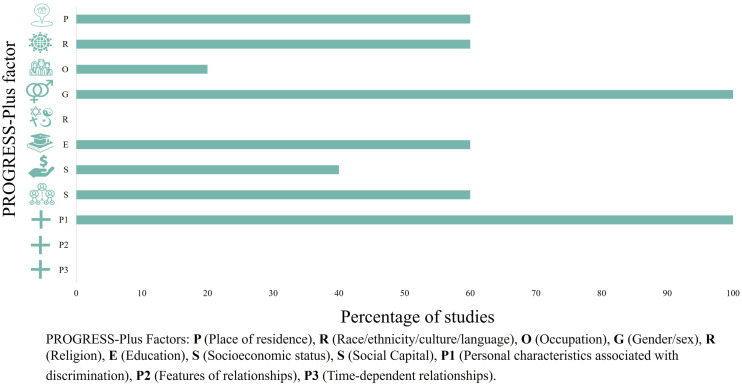
PROGRESS-Plus factors collected and reported in studies.

We summarize the reporting of PROGRESS-Plus factors in the identified studies here and in Figure 2, in the order of the framework.

**(1) Place of residence**: The residence location of participants was stated in three studies^15,19,20^ and reporting varied from considering the general geographic region of the selected clinics to hospital location. Studies recruited from various centres across Canada but were primarily conducted in British Columbia, Ontario, and Quebec.
**(2) Race, culture, ethnicity, and language:** Though often used interchangeably, the PROGRESS-Plus framework considers race as both a biological and social construct that is often linked to one’s physical traits and identity, whereas ethnicity is often linked to one’s national identity and implies a shared origin.^3^ Race was reported (unclear whether self-reported or assigned by investigators in four studies),^16–19^ with White participants ranging from 72.4% to 84%, but there were no specific considerations of related factors of culture, ethnicity, or language in any included studies. Two studies reported collecting ethnicity,^16,18^ but based on the definition they were actually reporting race. Of the studies reporting race, only one^18^ explicitly provided race categories other than ‘Caucasian’ or ‘White’ (specifically they included the terms Black, Asian, American Indian, and Other); however, there was no definition of what these categories encompass.
**(3) Occupation:** Occupation is typically used to refer to one’s profession; however, for this review we considered reporting of employment status to be relevant, particularly due to the overlap with SES and education. Employment status was reported in one study.^18^ The categories for occupation included Disabled, Homemaker, Working, Retired, or Student, with most participants working (55.4%).
**(4) Gender and/or sex:** Gender and sex were the most frequently reported PROGRESS-Plus factors. Participants were predominantly female in all studies, ranging from 84.4% to 100%, with two studies only including female participants.^17,19^ Additionally, there was some conflation of gender and sex, specifically with using sex (i.e. females and males) and gender (i.e. man and woman) terms interchangeably. All studies were limited to binary categories, with no inclusion of sex or gender-diverse terms.
**(5) Religion:** Religion of participants was not reported in any included study.
**(6) Education:** Education was reported in three studies^17,18,20^ and was considered as the highest level attained by participants from high school to postgraduate training, percentage of participants with a university degree or trades certificate, or as mean years of education. In one study the most frequent level of education reported by participants was completed university (39.1%).^18^ Another study reported that participants mean years of education ranged from 13.6 to 14.2 years.^17^ The final study reported 59.4% of participants had either a university degree or trades certificate.^20^
**(7) SES**: SES is distinct as it is multi-factorial, comprising income, occupation, and education.^14^ Two studies addressed aspects of SES.^17,20^ Specifically, they mentioned participant income levels. In one study they measured income by a combined household income scale that ranged from 1 to 6 (only levels 3 and 4 were specified by authors, specifically, 3 = $21,000–30,000; and 4 = $31,000–40,000), and participants primarily reported a household income level of 4, ranging from $31,000–40,000.^17^ In the other study reporting income, they collected gross annual household income with categories ranging from <$12,000 to over $100,000, with an option for participants to decline response.^20^ Participants primarily reported a gross annual income of $60,001–$80,000.
(8) **Social capital:** Social capital includes features of social structures (level of trust and norms) which act as a resource to individuals and facilitate collective action. As marital status is an important social factor associated with risk factors for health, morbidity, and mortality,^21^ it can be considered a feature of social capital according to the PROGRESS-Plus framework. Marital status was reported in three studies,^17,18,20^ with categories of married and co-habiting/common-law, divorced, separated, single, and widowed. Most participants were married; with one study reporting 59.4% of SLE participants were married or common-law,^20^ another reporting 41.9% were married,^17^ and the final study reporting 65.4% of participants were married or common-law.^18^
(9) **PLUS**: Finally, there was limited reporting of ‘Plus’ factors, that is, Personal characteristics associated with discrimination, Features of relationships, and Time-dependent relationships in included studies. Regarding personal characteristics associated with discrimination, age was reported across all studies. Reporting was continuous, (i.e. mean, standard deviation, and/or confidence intervals) in all studies, and participants were primarily middle aged, ranging from 42 years to 52.3 years. Features of relationships refer to those where one’s relationship with another has an impact on their own health (e.g. having parents who smoke), whereas time-dependent relationships refer to situations where an individual may temporarily be at a disadvantage (e.g. leaving a short-term care facility). Neither was addressed by the included studies.

## Discussion

We conducted a scoping review on intervention trials for people with SLE conducted in Canada to better understand how (or whether) determinants of health inequities are reported. Of 6 studies identified, all reported the sex and age distribution of the participants. The participants were generally middle-aged, female, and White. The next most reported determinants were place of residence, race, education, and social capital.

Our findings highlight three key issues. First, there is limited reporting of determinants of health inequities in RCTs of SLE in Canada to date. As aforementioned, sex and age were the only PROGRESS-Plus factors consistently reported across all 6 studies; other factors were either reported to a limited degree or not reported at all. This is problematic as SLE is a chronic disease, estimated to affect one in 2000 Canadians,^22^ requiring significant resources (financial and healthcare supports) in its treatment. For example, one study conducted out of Alberta, Canada found that the mean direct cost incurred by an individual with SLE is about $8000 CAD per year.^23^ This is about $2500 CAD more than mean cost estimates for an individual with rheumatoid arthritis ($5500 CAD) per year.^24^ This cost is in part due to medications, and coverage varies based on individual private insurance and provincial coverage as Canada does not have a national Pharmacare plan. Coverage is important to consider because in settings where there is no universal coverage for much of the population, the impact on healthcare access is heightened. In a cohort study in California, over 25% of participants reported medication cost concerns, and these concerns were associated with worse patient-reported outcomes.^25^ Treatment approaches in SLE are therefore significantly influenced by income level (which is linked to education and occupation), and these factors must be considered when evaluating interventions. Other relevant considerations are the amount of social support patients have, as chronic conditions present a significant amount of stress. A Canadian study found that females diagnosed with SLE before the age of 30 were less likely to be married/living common-law than expected based on general population rates, suggesting that sex and age can put patients more at risk of experiencing negative social effects (i.e. family relationships) of SLE.^26^ Furthermore, several studies have shown that SLE disproportionally affects minorities, with studies in the US citing that Black patients have earlier onset and more severe SLE.^27,28^ In Canada, research has shown that First Nations females have twice the prevalence of SLE compared to non–First Nations females.^7^ Due to this unequal distribution of SLE, it is therefore imperative that the diversity of the population is well represented in research cohorts to ensure that research outcomes are relevant to the typical population affected by these conditions. Without adequate reporting of these factors however, it is difficult to assure this.

The second issue is the lack of standardization in categorization. For example, when collecting race data, the reporting options differed in each study (e.g. White vs Caucasian); White refers to a race category while Caucasian refers to ethnicity. A distinction should be made in the reporting of the two, as an individual may be considered White racially but not Caucasian (or vice versa). This is an issue because without standardization it is challenging to compare and contrast different studies with respect to inclusion of different population groups. Having clearly defined race and/or ethnicity categories can help ensure that different racial and ethnic groups are well represented across research trials.

The final, and perhaps most significant, issue is that our findings demonstrate a lack of representation in clinical trials within Canada. As aforementioned, those who are middle-aged, female, and White are more likely to be represented. Given that research shows that minorities are disproportionately affected by SLE, this lack of representation is concerning. Issues with representation in SLE trials have also been recognized in other countries, such as the US, where there remain challenges enrolling participants from different populations.^6,11^ Sheikh et al. identified 5 key barriers to enrolment of diverse patients in SLE trials, including access, opportunity, health literacy, culture, and mistrust.^6^ These barriers should be considered when trying to improve representation and enrolment of diverse participants for SLE trials in Canada. Furthermore, researchers have suggested that guidance, transparency, and consensus with regulatory agencies are necessary to ensure future SLE studies reflect the true population living with the disease.^29^ A key example of this was the lack of racial and ethnic representation in belimumab clinical trials in the US, which led to mixed findings for Black patients, as there were insufficient numbers to conclude safety. As a result, the FDA initiated the Efficacy and Safety of Belimumab in Black Race Patients with SLE (EMBRACE) trial, where they were able to conclude that belimumab was safe for Black patients.^12^ Ultimately, without adequate representation in clinical trials, we are unable to determine whether treatments are effective for different populations, and thus appropriate measures should be taken to improve representation in SLE trials within Canada.

### Limitations

There are limitations associated with this scoping review. Studies were limited to RCTs as they are instrumental for identifying and evaluating effective therapies; however, this may have led to either underestimating or overestimating the true extent of health inequities in Canada, as we did not include other study designs. Further, RCTs may be limited in their capacity to measure determinants of inequity (e.g. have comprehensive questionnaires) and time for participants to complete such measures. Future studies to replicate our findings in reviews of observational studies and point-of-care trials to address determinants of equity would be beneficial. Finally, we used the PROGRESS-Plus framework to guide extraction of reporting of determinants of inequities; however, this framework may not be exhaustive, as other factors may be relevant that may not be captured within PROGRESS-Plus (e.g. social exclusion/inclusion, income distribution, neighbourhood safety, and infrastructure). However, PROGRESS-Plus is a leading framework for reporting health inequities and allowed us to formally consider how Canadian RCTs for SLE collect and report these factors.

## Conclusion

Determinants of inequities are not commonly reported or measured in Canadian RCTs for SLE interventions, which may present significant problems in the translation of findings to improved and personalized care. More importantly, where we could determine based on the available reporting, there remains a lack of representation in participants of SLE trials. Ongoing and future SLE research in Canada should carefully reconsider engagement practices, design and reporting to address health inequities introduced by standard practices.

## Supplemental Material

Supplemental Material - Reporting of determinants of health inequities and participant characteristics in randomized controlled trials of systemic lupus erythematosus in Canada: A scoping review Supplemental Material for Reporting of determinants of health inequities and participant characteristics in randomized controlled trials of systemic lupus erythematosus in Canada: A scoping review by Megan Thomas, Vanay Verma, Niloofar Gheshlagi, John Esdaile, Antonio Avina-Zubieta, Cheryl Barnabe, Mark Harrison, and Mary A De Vera in Lupus

## References

[1] Barnabe C . Disparities in rheumatoid arthritis care and health service solutions to equity. Rheum Dis Clin North Am 2020; 46(4): 685–692. DOI: 10.1016/j.rdc.2020.07.005. 32981645

[2] Williams JN Drenkard C Lim SS . The impact of social determinants of health on the presentation, management and outcomes of systemic lupus erythematosus. Rheumatology 2023; 62(Supplement_1): i10–i14. DOI: 10.1093/rheumatology/keac613. 36987604 PMC10050938

[3] O'Neill J Tabish H Welch V , et al. Applying an equity lens to interventions: using PROGRESS ensures consideration of socially stratifying factors to illuminate inequities in health. J Clin Epidemiol 2014; 67(1): 56–64. DOI: 10.1016/j.jclinepi.2013.08.005. 24189091

[4] Fatoye F Gebrye T Svenson LW . Real-world incidence and prevalence of systemic lupus erythematosus in Alberta, Canada. Rheumatol Int 2018; 38(9): 1721–1726. DOI: 10.1007/s00296-018-4091-4. 29987494 PMC6105153

[5] Bernatsky S Joseph L Pineau CA , et al. A population-based assessment of systemic lupus erythematosus incidence and prevalence--results and implications of using administrative data for epidemiological studies. Rheumatology (Oxford) 2007; 46(12): 1814–1818. DOI: 10.1093/rheumatology/kem233. 18032538

[6] Sheikh SZ Wanty NI Stephens J , et al. The state of lupus clinical trials: minority participation needed. J Clin Med 17; 8(8): 1245. DOI: 10.3390/jcm8081245. 31426523 PMC6722692

[7] Barnabe C Joseph L Belisle P , et al. Prevalence of systemic lupus erythematosus and systemic sclerosis in the First Nations population of Alberta, Canada. Arthritis Care Res 2012; 64(1): 138–143, DOI: 10.1002/acr.20656. 21972194

[8] DeQuattro K Yelin E . Socioeconomic status, health care, and outcomes in systemic lupus erythematosus. Rheum Dis Clin North Am 2020; 46(4): 639–649. DOI: 10.1016/j.rdc.2020.07.004. 32981641

[9] Carter EE Barr SG Clarke AE . The global burden of SLE: prevalence, health disparities and socioeconomic impact. Nat Rev Rheumatol 2016; 12(10): 605–620. DOI: 10.1038/nrrheum.2016.137. 27558659

[10] Peschken CA . Health disparities in systemic lupus erythematosus. Rheum Dis Clin North Am 2020; 46(4): 673–683, DOI: 10.1016/j.rdc.2020.07.010. 32981644

[11] Falasinnu T Chaichian Y Bass MB , et al. The representation of gender and race/ethnic groups in randomized clinical trials of individuals with systemic lupus erythematosus. Curr Rheumatol Rep 2018; 20(4): 20. DOI: 10.1007/s11926-018-0728-2. 29550947 PMC5857270

[12] Ginzler E Guedes Barbosa LS D'Cruz D , et al. Phase III/IV, randomized, fifty-two-week study of the efficacy and safety of belimumab in patients of Black African ancestry with systemic lupus erythematosus. Arthritis Rheumatol 2022; 74(1): 112–123. DOI: 10.1002/art.41900. 34164944 PMC9300099

[13] Gheshlaghi N Thomas M Trehan N , et al. Reporting of determinants of health inequities and participant characteristics in randomized controlled trials of juvenile idiopathic arthritis in Canada: a scoping review. Pediatric Rheumatology 2023; 21(1): 134. DOI: 10.1186/s12969-023-00917-5. 37932754 PMC10629131

[14] Thomas M Harrison M De Vera MA . Reporting of determinants of health inequities in rheumatoid arthritis randomized controlled trials in Canada: a scoping review. Arthritis Care Res 2023; 75(1): 101–114. DOI: 10.1002/acr.24978. 35792665

[15] Canadian Hydroxychloroquine Study Group . A randomized study of the effect of withdrawing hydroxychloroquine sulfate in systemic lupus erythematosus. N Engl J Med 1991; 324(3): 150–154. DOI: 10.1056/nejm199101173240303. 1984192

[16] Fortin PR Abrahamowicz M Ferland D , et al. Steroid-sparing effects of methotrexate in systemic lupus erythematosus: a double-blind, randomized, placebo-controlled trial. Arthritis Rheum 2008; 59(12): 1796–1804. DOI: 10.1002/art.24068. 19035431

[17] Edworthy SM Dobkin PL Clarke AE , et al. Group psychotherapy reduces illness intrusiveness in systemic lupus erythematosus. J Rheumatol 2003; 30(5): 1011–1016. 12734897

[18] Fortin PR Neville C Julien A-S , et al. Measuring the impact of MyLupusGuide in Canada: results of a randomized controlled study. Arthritis Care Res 2023; 75(3): 529–539, DOI: 10.1002/acr.24871. 35225436

[19] Moura CS Li R Lawrie S , et al. Humor in systemic lupus erythematosus. Eur J Rheumatol 2015; 2(1): 5–9. DOI: 10.5152/eurjrheumatol.2015.0070. 27708912 PMC5047248

[20] Li LC Feehan LM Xie H , et al. Efficacy of a physical activity counseling program with use of a wearable tracker in people with inflammatory arthritis: a randomized controlled trial. Arthritis Care Res 2020; 72(12): 1755–1765. DOI: 10.1002/acr.24199. 32248626

[21] Lindström M . Marital status, social capital, material conditions and self-rated health: a population-based study. Health Policy 2009; 93(2): 172–179. DOI: 10.1016/j.healthpol.2009.05.010. 19692141

[22] Systemic lupus erythematosus. 2017. https://arthritis.ca/about-arthritis/arthritis-types-(a-z)/types/systemic-lupus-erythematosus

[23] Fatoye F Gebrye T Svenson LW . Direct health system costs for systemic lupus erythematosus patients in Alberta, Canada. PLoS One 2021; 16(5): e0251409. DOI: 10.1371/journal.pone.0251409. 33961687 PMC8104382

[24] Ohinmaa AE Thanh NX Barnabe C , et al. Canadian estimates of health care utilization costs for rheumatoid arthritis patients with and without therapy with biologic agents. Arthritis Care Res 2014; 66(9): 1319–1327. DOI: 10.1002/acr.22293. 24470178

[25] Aguirre A DeQuattro K Shiboski S , et al. Medication cost concerns and disparities in patient-reported outcomes among a multiethnic cohort of patients with systemic lupus erythematosus. J Rheumatol 2023; 50: 1302–1309, jrheum. DOI: 10.3899/jrheum.2023-0060 37321640 PMC10543599

[26] Brailovski E Vinet E Pineau CA , et al. Marital status and age of systemic lupus erythematous diagnosis: the potential for differences related to sex and gender. Lupus Sci Med 2019; 6(1): e000325. DOI: 10.1136/lupus-2019-000325. 31448125 PMC6687032

[27] Hasan B Fike A Hasni S . Health disparities in systemic lupus erythematosus-a narrative review. Clin Rheumatol 2022; 41(11): 3299–3311. DOI: 10.1007/s10067-022-06268-y. 35907971 PMC9340727

[28] Williams EM Ortiz K Browne T . Social determinants of health, the chronic care model, and systemic lupus erythematosus. Int J Chronic Dis 2014; 2014: 361792. DOI: 10.1155/2014/361792. 26464854 PMC4590929

[29] Sheikh SZ Englund TR Burriss SW , et al. EMBRACE: one small story in lupus—one giant challenge in clinical trials. ACR Open Rheumatol 2022; 4(9): 747–752, DOI: 10.1002/acr2.11477. 35748175 PMC9469483

