# Supplementary material for: Reporting of determinants of health inequities and participant characteristics in randomized controlled trials of systemic lupus erythematosus in Canada: A scoping review
Source: Lupus. 2024 Feb 9;33(5):462–9. doi: 10.1177/09612033241233032 (PMC10955787; doi:10.1177/09612033241233032)
Supplement: Supplemental Material - Reporting of determinants of health inequities and participant characteristics in randomized controlled trials of systemic lupus erythematosus in Canada: A scoping review [file sj-pdf-1-lup-10.1177_09612033241233032.pdf]

Supplementary Materials: Search Strategies

MEDLINE (Ovid) search strategy

Ovid MEDLINE(R) and Epub Ahead of Print, In-Process, In-Data-Review & Other Non-Indexed Citations, Daily and Versions(R) <1990 to June 2023>

Line number	Search terms
1	lupus.mp. or Lupus Erythematosus, Cutaneous/ or Lupus Nephritis/ or Lupus Erythematosus, Systemic/ or Lupus Erythematosus, Discoid/ or Lupus Vasculitis, Central Nervous System/
2	randomized controlled trial.pt.
3	controlled clinical trial.pt.
4	randomized.ab.
5	placebo.ab.
6	drug therapy.fs.
7	randomly.ab.
8	trial.ab.
9	groups.ab.
10	or/2-9
11	exp animals/ not humans.sh.
12	10 not 11
13	1 and 12
14	exp Canada/
15	(canad* or british columbia or alberta or saskatchewan or manitoba or ontario or quebec or new brunswick or prince edward island or nova scotia or newfoundland or labrador or nunavut or northwest territories or yukon).af.
16	14 or 15
17	13 and 16
18	limit 17 to yr="1990 -Current"

Embase (Ovid) search strategy

Embase <1990 to June 2023>

Line number	Search terms
1	Lupus Erythematosus, Systemic/ or Lupus Erythematosus, Systemic.mp.
2	Lupus Nephritis/ or Lupus Nephritis.mp.
3	Lupus Vasculitis, Central Nervous System/ or Lupus Vasculitis, Central Nervous System.mp.
4	lupus.mp.
5	or/1-4
6	random$.tw.
7	factorial$.tw.
8	crossover$.tw.
9	cross over.tw.
10	cross-over.tw.
11	placebo$.tw.
12	(doubl$ adj blind$).tw.
13	(single$ adj blind$).tw.
14	assign$.tw.
15	allocat$.tw.
16	volunteer$.tw.
17	crossover procedure/
18	double blind procedure/
19	randomized controlled trial/
20	single blind procedure/
21	or/6-20
22	5 and 21
23	exp Canada/
24	(canad* or british columbia or alberta or saskatchewan or manitoba or ontario or quebec or new brunswick or prince edward island or nova scotia or newfoundland or labrador or nunavut or northwest territories or yukon).af.
25	23 or 24
26	22 and 25
27	limit 26 to yr="1990 -Current"

Cochrane Central Register of Controlled Trials (CENTRAL) (via Ovid) search strategy

EBM Reviews - Cochrane Central Register of Controlled Trials <June 2023>

Line number	Search terms
1	Lupus Vulgaris/ or Lupus Erythematosus, Cutaneous/ or Lupus Nephritis/ or Lupus Erythematosus, Systemic/ or lupus.mp. or Lupus Erythematosus, Discoid/ or Lupus Vasculitis, Central Nervous System/
2	exp Canada/
3	(canad* or british columbia or alberta or saskatchewan or manitoba or ontario or quebec or new brunswick or prince edward island or nova scotia or newfoundland or labrador or nunavut or northwest territories or yukon).af.
4	2 or 3
5	1 and 4
6	limit 5 to yr="1990 -Current"
